# Deep-Learning-Based Prediction of High-Risk Taxi Drivers Using Wellness Data

**DOI:** 10.3390/ijerph17249505

**Published:** 2020-12-18

**Authors:** Seolyoung Lee, Jae Hun Kim, Jiwon Park, Cheol Oh, Gunwoo Lee

**Affiliations:** 1 Research Institute of Engineering Technology, Hanyang University Erica Campus, Ansan 15588, Korea; lsy0717@hanyang.ac.kr (S.L.); jhkim8182@hanyang.ac.kr (J.H.K.); 2 Department of Transportation and Logistics Engineering, Hanyang University Erica Campus, Ansan 15588, Korea; wldnjsdm@hanyang.ac.kr (J.P.); cheolo@hanyang.ac.kr (C.O.)

**Keywords:** artificial neural network, deep learning, traffic safety, taxi driver wellness, random forest method

## Abstract

Background: Factors related to the wellness of taxi drivers are important for identifying high-risk drivers based on human factors. The purpose of this study is to predict high-risk taxi drivers based on a deep learning method by identifying the wellness of a driver, which reflects the personal characteristics of the driver. Methods: In-depth interviews with taxi drivers are conducted to collect wellness data. The priorities of factors affecting the severity of accidents are derived through a random forest model. In addition, based on the derived priority of variables, various combinations of inputs are set as scenarios and optimal artificial neural network models are derived for each scenario. Finally, the model with the best performance for predicting high-risk taxi drivers is selected based on three criteria. Results: A model with variables up to the 16th priority as inputs is selected as the best model; this has a classification accuracy of 86% and an F1-score of 0.77. Conclusions: The wellness-based model for predicting high-risk taxi drivers presented in this study can be used for developing a taxi driver management system. In addition, it is expected to be useful when establishing customized traffic safety improvement measures for commercial vehicle drivers.

## 1. Introduction

The number of deaths from traffic accidents is decreasing. However, according to the data on the reduction rate of deaths in traffic accidents by vehicle type in the last 10 years, the reduction rate of noncommercial vehicles was 39%, while that of commercial vehicles was 31% [1]. Efforts to improve the risk of accidents in business vehicles are essential for zero traffic accident deaths [2,3]. According to the dataset of commercial vehicles’ traffic accidents in 2019 in Korea, taxis were involved in 40.1% of commercial vehicle accidents. In addition, among fatalities involving commercial vehicles, 25% involved a taxi; this demonstrates the need for social countermeasures to reduce traffic accidents [3]. In a review of the existing literature related to the traffic safety of commercial vehicles, it was found that a driver’s working environment, personality, fatigue, and mental and physical health are the main factors influencing traffic safety [4,5]. In particular, taxi drivers have high levels of fatigue and stress because they work for long shifts, and these conditions affect their traffic safety records [6,7,8]. More specifically, these poor working environments and mental conditions can result in drowsy driving and dangerous driving, which lead to traffic accidents [9,10,11,12].

Although there have been various efforts to improve the quality of life and working environments of taxi drivers, continuous management plans for taxi drivers to reduce accidents are still insufficient. As human factors account for about 94% of the major reasons of traffic accidents, it is essential to understand and manage drivers’ intrinsic factors to reduce traffic accidents [13]. Therefore, in order to prevent traffic accidents related to taxis, it is necessary to identify high-risk taxi drivers based on human factors. In this study, the concept of wellness is applied to investigate the human factors of taxi drivers and present a methodology reflecting these factors to prevent traffic accidents. Wellness is related to well-being and health, and in a modern society, the meaning has been expanded to include happiness [14,15]. Wellness implies a healthy state physically, mentally, and socially. In this study, it is defined as a concept reflecting the working environment, living environment, and health characteristics of taxi drivers. Factors related to the wellness of taxi drivers are important for identifying high-risk drivers based on human factors, and they are also essential for preparing customized traffic safety measures that reflect individual characteristics.

The purpose of this study is to predict high-risk taxi drivers based on a deep learning method using wellness data. This study consists of Stage 0 through Stage 3. Stage 0 is the data construction phase. In this phase, in-depth interviews are conducted to identify the wellness of commercial vehicle drivers, and this wellness data is matched with an accident dataset collected from the commercial vehicle driver management system operated by the Korea Transport Safety Authority (KOTSA). In Stage 1, the priority of factors affecting the severity of accidents is defined, and the random forest analysis is applied as the methodology. Stage 2 involves constructing an optimized model based on an artificial neural network (ANN), which classifies the severity level of an accident. In this phase, by using the priority of variables derived from Stage 1, combinations of input data are set as scenarios, and optimized classifiers for each scenario are derived. Stage 3 aims to choose an optimized model for predicting high-risk taxi drivers. In this phase, the selection criteria for the optimized model are established and applied. This derived model is expected to be useful in determining high-risk taxi drivers. With the model, it should be possible to support customized consulting systems for commercial vehicle drivers and manage wellness factors that are highly related to accidents. This study can be used to induce active improvements in traffic safety through drivers’ self-diagnosis and feedback.

The rest of this paper is organized as follows. Section 2 presents the analysis methodology and mentions the overall research flow, data preparation, and principles of model construction of the random forest and ANN, which are the deep learning techniques used in this study. In Section 3, the analysis results are presented in three steps; the first step derives the priority of factors affecting accident severity based on the random forest model, the second derives a classifier through ANN optimization, and the third presents the optimized model selection criteria and the best model for predicting a high-risk taxi driver. Finally, Section 4 discusses the conclusions and future research directions based on the identified limitations of this study.

## 2. Methodology

**Figure 1:**
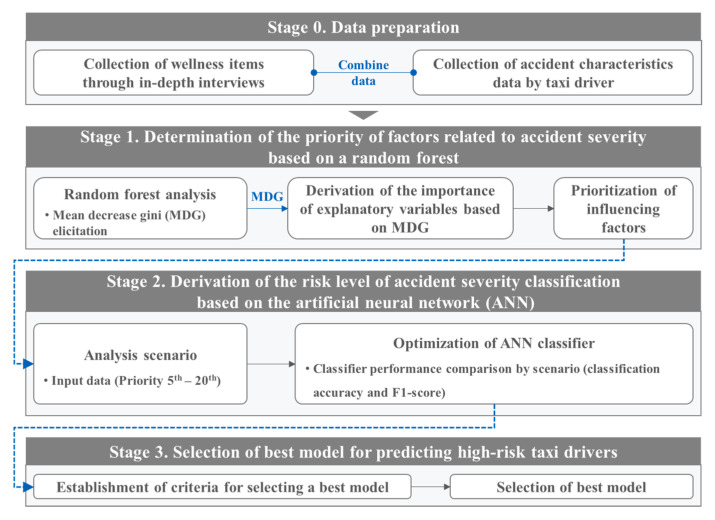
Overall research framework.

In this study, a deep learning method is applied to predict high-risk taxi drivers through driver wellness evaluation, and the process of the study is presented in Figure 1. The study consists of multiple stages. In Stage 0, wellness items are collected through an in-depth interview; this information is matched with the commercial vehicle driver’s accident data. In Stage 1, the priority of factors affecting the severity of accidents is derived, and a random forest model is applied as the analysis model. In Stage 2, different priorities of factors are applied to create scenarios for the ANN classifiers, and the optimal ANN classifiers that predict the severity of accidents are derived for each scenario. Finally, in Stage 3, the best model for predicting high-risk taxi drivers is selected by considering the classification accuracy and the number of input data.

### 2.1. Data Preparation

As of July 2020, there were 254,490 taxi drivers in Korea, of which 89,650, or 35%, belonged to taxi companies. Moreover, private taxi drivers account for 65% of taxi drivers, and ride-hailing services such as Uber do not operate in Korea. In Stage 0, to identify the wellness of taxi drivers, in-depth interviews were conducted by a professional investigator. In the in-depth interviews, professional investigators were matched one-to-one to increase the reliability of the data. The subjects of the in-depth interviews were drivers belonging to taxi companies; these drivers caused serious traffic accidents from the third quarter of 2018 to the second quarter of 2019. Drivers belonging to taxi companies (rather than individual taxi drivers) were selected as the subject of the survey because corporate taxi companies in which serious accidents occurred are obligated to undergo safety inspections and have high access to investigations. Interviews were conducted with 993 drivers at 89 taxi companies from September to October 2019. The survey items consist of 246 variables, including wellness, the characteristics of the driver, and company data. The 20 wellness variables related to traffic safety were selected based on the literature review presented in Table 1.

The investigation items consist of 20 categories related to wellness and traffic safety, which were collected through the interviews, and one accident characteristic factor, which was collected through the commercial vehicle driver management system. The items and scale of the survey are presented in Table 2. The wellness categories include a worker’s working environment (level of satisfaction, working hours per week), living environment (level of satisfaction), and health characteristics (sleeping time, level of stress). If the survey scale is 5 points, it represents the level of the question: ‘Strongly disagree’, ‘Disagree’, ‘Neutral’, ‘Agree’, and ‘Strongly agree.’ In order to examine the validity of the wellness data, the presence of missing values and the same response rate are identified. As a result, it is found that there are no missing values in all 20 items, and all items are valid with the same response rate within 90%. Furthermore, based on the correlation analysis with 20 variables and the number of accidents, the correlation between accidents and all variables was statistically significant.

In order to analyze the accident characteristics of the interviewed taxi drivers, we used the dataset of the number of casualties per traffic accident from August 2016 to July 2019; this was obtained from the commercial vehicle driver management system. Accident data were also considered only when the taxi driver was the perpetrator. Among the 993 interviewed drivers, accident data were collected for 781 drivers. From the collected data, the accident severity is classified into three levels based on the severity of the accident, and this is defined as the risk level of accident severity. High-risk (class 3) refers to a driver who has experienced more than a serious injury, medium-risk (class 2) refers to a driver who has experienced a minor injury, and low-risk (class 1) refers to a driver with no accident experience. The numbers of drivers for the different risk levels of accident severity are 66, 164, and 551, respectively.

### 2.2. Random Forest Model

The random forest model is an ensemble machine learning technique, and it uses an advanced decision tree analysis methodology to overcome the drawbacks of decision tree analysis [26]. In the random forest model, every tree is created in the learning process based on bootstrap samples that are randomly selected with replacements. The number of trees is set by the analyst in advance, and the average values of the results for each tree are derived as the final outputs, based on the results generated in each tree. Random forest models are a technique that can build a model with excellent predictive performance. Compared to a decision tree, which has a high probability of overfitting, a random forest has the advantage of supplementing the limitations of overfitting from the decision tree [26]. The process of random forest learning using bootstrap sampling proceeds according to the following steps: (i) Generate the trees and training datasets from the specified training dataset by sampling the bootstrap, (ii) train a basic tree sorter, (iii) combine the basic sorter (tree) into one sorter (random forest), and (iv) derive the final prediction results by the majority voting rule. Observed values in the random forest that are not included in the learning cycle of individual trees are considered out-of-bagging (OOB) and are used to validate the model. OOB is used as the category to estimate the predicted odds and to classify the variables that cause anomalies. The number of times that OOB is chosen in all random forest decision trees varies for each tree, and the expected values are different for each tree. The probability of predicting the OOB observation correctly for each observation in the original category, which belongs to category *k*, is calculated using Equation (1).

(1) $$\hat{p_{k}}\left(x_{i}\right)=\frac{\sum j\in \bar{OOB_{i}}-I\left[\hat{y}\left(x_{i},t_{j}\right)=k\right]}{\left|OOB_{i}\right|},\;\mathrm{for}\;k$$

Here, *i* is an indicator function that is set as 1 when the value in the parentheses is true and set to 0 when the value is false. Additionally, $\hat{y}\left(x_{i},t_{j}\right)$ represents the predicted category and *t_j_* means the *j*th decision tree in the generated decision tree (*t*).

*OOB_i_* is a group of decision trees that is not used in the learning process, by bagging as an observed attribute. If a set of decision trees does not include $x_{i}$, the ratio of the number of decision trees predicting $x_{i}$ to the k category is $\hat{p_{k}}\left(x_{i}\right)$ [27]. The verification method using OOB is as accurate as the method through new verification data. It indicates that there is no need to configure a separate test set when measuring OOB [26,27]. This study uses the mean decrease Gini (MDG) as an indicator to measure the importance of explanatory variables in random forests. The MDG is the average reduction in the GI index for a given explanatory variable in all trees. If the number of classification categories (*i*) is *j*, that is, i=1, 2, 3, ⋯, J, the GI index is calculated as shown in Equation (2).

(2) $$\mathrm{GI}=\sum_{i=1}^{J}f_{i}\left(1-f_{i}\right)=1-\sum_{i=1}^{J}f_{i}^{2}$$

Here, *f_i_* is the ratio of classifying *i* to the *i* category correctly, and 1 − *f_i_* is the ratio of classifying *i* to another category. If the model perfectly classifies every category, *f_i_* is 1 and the value of the GI index becomes 0. A higher MDG value for a particular variable indicates that this value is suitable for correct classification of a certain category, meaning that it decreases the degree of impurity. The MDG value varies from 0 to 100. When an MDG value of one variable is 0, the variable will not be used for classification. However, if the MDG is closer to 100, the observation can be completely classified by the variable.

### 2.3. Artificial Neural Networks (ANN)

An ANN is a data processing system resulting from the mathematical modeling of the learning process inspired by humans. It consists of an input layer that accepts input data, a hidden layer that processes input values and produces the result, and an output layer that calculates an output value [28]. Each layer is composed of nodes, and results are derived from the linkage between the nodes and the action of the transfer function.

The pattern recognition network applied in this study is a feed-forward network, which can be trained to classify inputs according to the classes of outputs. The feed-forward network is a method in which the signal from the input layer is forwarded to the hidden layer and the signal from the hidden layer is forwarded to the output layer. The hyperparameters that need to be optimized in neural networks are the number of hidden layers and neurons, as well as the transfer function that calculates the output value of the neurons. The descriptions of each hyperparameter are shown in Table 3.

For neural network optimization, some studies have applied the Bayesian optimization method, which effectively solves the global optimization problem [31,32,33]. Bayesian optimization is a methodology for tuning hyperparameters, finding the value of *x* that maximizes the objective function *f(x)*. This is defined as shown in Equation (3) [34]. This study defines the correct classification rate (CCR) of the classifier as the objective function and derives a hyperparameter x that maximizes CCR.

(3) $$x^{*}=argmax_{x}f\left(x\right)$$

where

- x*: Optimized hyperparameters
- *x*: Hyperparameter
- *f*(*x*): Correct classification rate (CCR) of the models.

Bayesian optimization constructs a probabilistic model for *f(x)*. This process is outlined as follows: (i) With the assumption that *f(x)* follows the Gaussian process (GP) prior, learn the model by using the given data $D\left(=\left\{\left(x_{1},\;f\left(x_{1}\right)\right),\left(x_{2},\;f\left(x_{2}\right)\right),\;\cdots ,\;\left(x_{n},\;f\left(x_{n}\right)\right)\right\}\right)$, (ii) calculate the acquisition function for data not included in *D*, and (iii) include the data point $\left(x_{n},\;f\left(x_{n}\right)\right)$ in *D* that has the largest acquisition function value. The acquisition function is a measure used to find the global optimum, which is the hyperparameter affecting the maximum classification accuracy. In this study, the expected improvement (EI) function is selected as the acquisition function. EI minimizes the error of the predicted *f*(*x*), and it is defined as shown in Equation (4) [31,35,36,37]. For calculating the mean and standard deviations of predictions from the model, the Gaussian process (GP) is used. The GP is suitable for the Bayesian optimization algorithm because it facilitates incremental learning and variance calculation for predicted values [38,39,40].

(4) $$\begin{array}{c} z=\frac{\hat{f}{\left(x\right)}_{max}-\mu \left(\hat{f}\left(x\right)\right)}{\sigma \left(\hat{f}\left(x\right)\right)}\\ EI\left(x\right)=\sigma \left(zG\left(z\right)+g\left(z\right)\right) \end{array}$$

- $\hat{f}{\left(x\right)}_{max}$: Maximum of CCR predicted for hyperparameters
- $\mu \left(\hat{f}\left(x\right)\right)$: Average of CCR predicted for hyperparameters
- $\sigma \left(\hat{f}\left(x\right)\right)$: Standard deviation of CCR predicted for hyperparameters
- (*G*)*z*: Normal cumulative distribution function
- (*g*)*z*: Probability density function for *z*.

## 3. Results and Discussion

### 3.1. Determination of the Priority of Factors Related to Accident Severity Based on a Random Forest (Stage 1)

**Figure 2:**
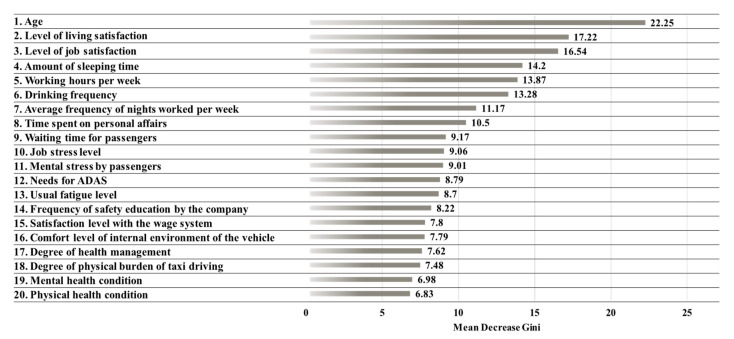
Results of the mean decrease Gini (MDG) by factors affecting the degree of accident severity.

The purpose of Stage 1 is to derive the priority of factors affecting the severity of accidents by using the random forest method. To do this, the risk level of accident severity is defined as the target variable and 20 wellness items are set as input variables. For training the random forest model, the total number of trees is optimized to 500, and it is derived as an optimization phase that builds trees with two randomly chosen variables when configuring each node. In the optimized model, the OOB error is shown as 30.94%. However, classification accuracy is not considered in this study, because the purpose of the random forest analysis is to derive the priority of factors affecting the severity of accidents. The importance of factors that affect the classification of a taxi driver’s accident severity risk level is evaluated based on the MDG value, which is the evaluation index for the importance of an input variable. The evaluation results are presented in Figure 2. The MDG of the driver’s age is shown as 22.25; this is determined to be the most effective factor in classifying the risk level of accident severity. In addition, the most important factors of classification are derived in the order of the average living satisfaction, level of job satisfaction, amount of sleeping time, and working hours per week. Combinations of five to twenty variables with the highest priority of factors, as derived in Stage 1, are used as the inputs of the ANN model in Stage 2.

### 3.2. Derivation of the Risk Level of Accident Severity Classification Based on the ANN (Stage 2)

Stage 2 involves deriving an optimized ANN model that classifies the risk level of accident severity. In this phase, the combinations of inputs with the highest priority, as derived from Stage 1, are developed. Sixteen combinations of inputs are set as scenarios. For example, in the case of a scenario that uses 10 variables, the 1st to 10th variables derived from Stage 1 are used as input variables. In each scenario, optimized ANN classifiers are derived. The ratio of training data to test data for the ANN model is defined as 7:3.

The classification accuracies and F1-scores for each scenario are shown in Table 4. The F1-score is a harmonized average of precision and recall that can accurately evaluate the model’s performance when the data label is imbalanced. The larger the F1-score, the better the model can be determined, and the calculation formula is presented in Equation (5) [41,42]. From Table 3, it can be seen that the average of the overall classification accuracy for all scenarios is 81%. Additionally, the averages of classification accuracies by risk level of accident severity are 94%, 52%, and 29%, corresponding to Classes 1, 2, and 3, respectively. In addition, the overall classification accuracy of the scenarios in which all 20 variables are used is found to be 87%, which is higher than other scenarios. In terms of the F1-score, the overall mean is found as 0.59, and the F1-score of the scenario in which 18 variables are used is 0.80, which is the highest score among the scenarios. As the scenarios with the best performance are different according to the performance criteria, it is found that criteria for selecting the best scenario are necessary.

$$F1\text{-}\mathrm{score}=2\times \frac{Precision\times Recall}{Precision+Recall}$$

(5) $$\text{Precision}=\frac{TP}{TP+FP}$$

$$\text{Recall}=\frac{TP}{TP+FN}$$

where

- *TP*: True positive
- *FP*: False positive
- *FN*: False negative.

### 3.3. Selecting the Best Model for Predicting High-Risk Taxi Drivers (Stage 3)

**Figure 3:**
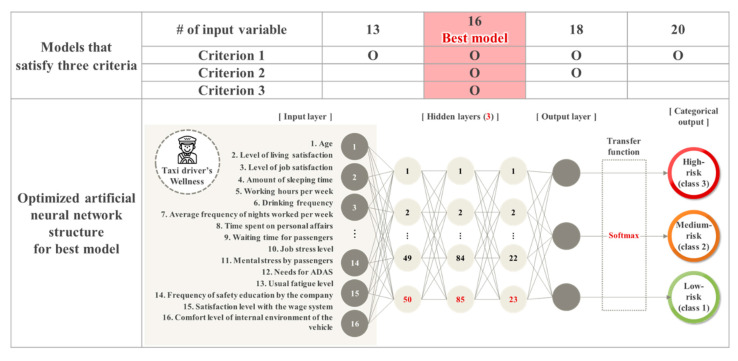
Results of selecting the best model.

From Stage 2, it is confirmed that the performances of the ANN model in each scenario are different. Therefore, in Stage 3, three criteria are designed to select the best model, as follows. Criterion 1 is based on the F1-score, and the 75th percentile value of 0.77 is set as the threshold for satisfying this criterion. Through criterion 1, a model considering the prediction performances of all classes can be determined. However, as the F1-score of the model is highly influenced by the classification accuracy of Class 1 (low-risk) with a large number of samples, criterion 1 is limited in that it does not accurately reflect the classification accuracy of Classes 2 (medium-risk) and 3 (high-risk). In order to address the limitation of criterion 1, the classification accuracies of the three classes are checked as the second criterion. In criterion 2, 53% is defined as the threshold, which is the 75th percentile value of the classification accuracy for Class 3, which shows the lowest average accuracy value. If the classification accuracies of each class are 53% or more, criterion 2 is satisfied. Finally, criterion 3 determines whether the model can show a similar performance with the minimum dataset. In this study, to expand and easily use the classification results in the future, it is expected that a model working with a minimal data collection would be practical. Accordingly, in criterion 3, models satisfying criteria 1 and 2 show similar performance. When the performances of the models are similar, the model with the smaller number of input variables is determined as the better model. A summary of these three criteria is shown below, and the models that satisfy these criteria are shown in Figure 3.

Criterion (1) The F1-score is 0.77 or higher.
Criterion (2) The classification accuracies of each class are 53% or higher.
Criterion (3) Scenarios that satisfy criteria 1 and 2, with fewer input variables.

As a result of selecting the best model according to the three criteria mentioned above, the model with 16 input variables is selected as the best model. Figure 3 shows the structure of the optimized model with 16 variables. Among the hyperparameters of the model, softmax is chosen as the transfer function. The number of hidden layers of the optimized model is found to be three, and the numbers of neurons per hidden layer are 50, 85, and 23, respectively. The F1-score of the model is 0.77, and the overall classification accuracy is 86%. In addition, the classification accuracies of each class are 90%, 83%, and 63%, respectively, showing higher performance than other models.

## 4. Discussion

**Figure 4:**
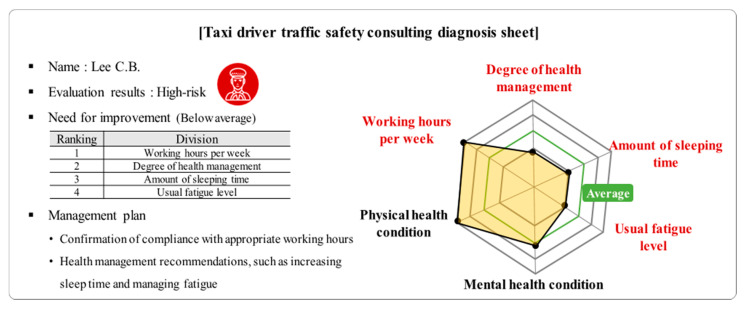
Examples of customized safety consulting approaches based on prediction model of high-risk taxi drivers.

Effective traffic accident prevention is possible when taxi drivers’ working environment, life patterns, and active management of health characteristics, and the degree of interest in taxi companies’ driver management, are improved. The analysis results of this study will be used as basic data to improve the effect of preventing traffic accidents in commercial vehicles. For example, the traffic safety manager of a taxi company may investigate 16 items derived from the analysis results of a taxi driver’s traffic-safety survey. It is possible to perform customized traffic safety consulting for taxi drivers using the factors derived from the best model. Figure 4 illustrates an example of a traffic safety consulting diagnosis result for taxi drivers. The driver presented in the example was diagnosed as a high-risk driver, and the working hours per week were higher than the average. In this case, ‘confirmation of appropriate working hours’ as a management plan can be presented. With the high-risk taxi driver prediction model proposed in this study, it is also possible to provide customized diagnosis results and establish a transportation safety consulting system. Such a system will help improve life patterns and the working environment by providing regular transportation safety consulting and diagnosis charts. Specific safety and health improvement plans can be prepared for each taxi company to reduce the number of high-risk taxi drivers. Furthermore, it can be used as basic data for establishing an evaluation and compensation system to encourage safe driving.

## 5. Conclusions

Taxis account for the largest share at 40.1% of the traffic accidents in commercial vehicles. Moreover, the reduction rate of deaths in taxi accidents was low compared to other commercial vehicles. Therefore, efforts should be made to improve the traffic safety of taxis by preparing effective measures. Many taxi drivers are exposed to overwork due to inadequate working environments and long working hours. Therefore, a traffic safety management system for taxis based on underlying human factors is required. However, existing studies investigating the relationship between accidents and drivers have focused on physical factors and demographic characteristics, such as accident sites and vehicle factors. Few studies have looked into intrinsic aspects of the drivers.

This study develops a risk level of accident severity classifiers to predict high-risk taxi drivers based on a deep learning method with wellness data. The study is broken into several stages. In Stage 0, wellness data are collected, including information related to drivers’ working environments, living environments, and health characteristics, through in-depth interviews conducted with 993 taxi drivers. In addition, high-risk drivers are classified based on the severity of the accidents they experienced, which is derived from the accident dataset of the drivers collected from a driver management system. High-risk drivers were classified based on the severity of the accidents they experienced. In Stage 1, a random forest analysis is used to identify the priorities of factors affecting the risk level of accident severity that taxi drivers experienced. As a result, a driver’s age, living satisfaction, level of job satisfaction, amount of sleeping time, and working hours per week are identified as the top five variables that have the greatest influence on the risk level of accident severity. In Stage 2, optimized ANN classifiers are derived to predict the risk level of accident severity for 16 scenarios using the priority of variables derived in Stage 1. Finally, in Stage 3, the best model for predicting high-risk taxi drivers is selected based on three criteria considering the classification accuracy, F1-score, and number of input variables. As a result, the scenario with input variables up to the 16th priority is selected as the best model; this showed a classification accuracy of 86% and an F1-score of 0.77. With the optimal model derived in this study, high-risk taxi drivers can be identified. Based on these results, it is expected that traffic safety measures can be established to reflect the wellness of individual drivers, which can be used to manage high-risk drivers.

In order to increase the reliability of the high-risk taxi driver classifier developed in this study, further studies should be undertaken in the future. First, this study identifies 20 items related to the wellness of taxi drivers through an interview, but it is necessary to consider more variables that can represent wellness, such as the job-related affective well-being scale (JAWS), physical symptoms inventory (PSI), and so on. In the case of health-related variables, the reliability of data can be improved through objective data collection, such as data from medical institutions. In addition, while this study predicts the risk level of accident severity, it is also necessary to consider other variables related to traffic safety, such as the number of accidents and dangerous driving behavior. Additionally, it is necessary to consider the driving distance and the number of working days as exposure to traffic accidents. Second, it is necessary to secure the reliability of the prediction model by collecting additional data. In order to increase the accuracy of the collected data, this study applies an interview method. However, face-to-face interviews take a lot of time. Therefore, a way to collect more data while increasing the accuracy of the response is necessary in the future. Furthermore, it is necessary to predict high-risk taxi drivers with consideration for the types of taxi drivers by expanding the survey groups to individual taxi drivers. Finally, we may be able to increase the reliability of the model by considering various deep learning methods, such as k-fold validation.

This study derives classifiers for predicting high-risk taxi drivers based on the driver’s wellness, which goes beyond the physical factors of traffic accidents. It is expected that the results of this study can be used to prepare plans for changing the paradigm of taxi traffic safety measures based on wellness. Additionally, it is also expected that these results can be used as basic data for establishing an effective traffic safety policy.

## Figures and Tables

**Table 1:** Review of existing literature for selection of wellness data related to traffic safety.

Literatures	Considered Factors
Hagl and Kouabenan [16] Staubach et al. [17]	Needs for advanced driver assistance system (ADAS)
Li et al. [18]	Working hours/Time spent on personal affairs/Fatigue level
Meng et al. [15]	Fatigue level
Wang et al. [9]	Level of job satisfaction
Wang et al. [9] Ba et al. [19]	Frequency of safety education by the company
Vahedi et al. [20]	Working hours per week/Level of living satisfaction/Age
Ishimaru et al. [21]	Drinking frequency
Bulduk et al. [22]	Usual fatigue level/Age
Lim et al. [23] Williamson et al. [24]	Amount of sleeping time
Burgel et al. [6]	Degree of health management/Satisfaction level with the wage system/Comfort level of internal environment of the vehicle
Di Milla et al. [25]	Physical health condition/Mental health condition/Waiting time for passengers
Raanaas et al. [7]	Mental stress by passengers/Degree of physical burden of taxi driving/Average frequency of nights worked per week
Chen et al. [8]	Job stress level/Level of job satisfaction

**Table 2:** The description of variables.

Division	Investigation Item	Survey Scale
In-depth interview	Mental stress by passengers	5 points
	Drinking frequency	6 points
	Degree of health management	5 points
	Physical health condition	5 points
	Mental health condition	5 points
	Usual fatigue level	5 points
	Amount of sleeping time	narrative
	Working hours per week	narrative
	Satisfaction level with the wage system	5 points
	Job stress level	5 points
	Needs for advanced driver assistance system (ADAS)	5 points
	Waiting time for passengers	5 points
	Level of job satisfaction	5 points
	Comfort level of internal environment of the vehicle	5 points
	Degree of physical burden of taxi driving	5 points
	Average frequency of nights worked per week	narrative
	Time spent on personal affairs	5 points
	Frequency of safety education by the company	5 points
	Level of living satisfaction	5 points
	Age	narrative
Driver management system	Risk level of accident severity (High-risk, Medium-risk, Low-risk)	3 categories

**Table 3:** Hyperparameters for neural network [29,30].

Hyperparameter		Description	
Transfer function	Softmax	Maps the nonnormalized output to a probability distribution over predicted output classes	$h\left(x\right)=\frac{\exp\left(x\right)}{\sum \exp\left(x\right)}$
Number of hidden layers		Number of hidden layers	
Number of neurons		Number of neurons in the hidden layers	

**Table 4:** Correct classification rate and F1-score by scenarios.

# of Input Variables	Priority of Factors by Stage 1	Class				F1-Score
		1 (Low-Risk)	2 (Medium-Risk)	3 (High-Risk)	Total	
5	1st–5th	96%	38%	0%	76%	0.47
6	1st–6th	100%	0%	0%	71%	0.28
7	1st–7th	90%	37%	53%	76%	0.61
8	1st–8th	96%	29%	32%	77%	0.58
9	1st–9th	97%	47%	11%	79%	0.61
10	1st–10th	95%	41%	0%	76%	0.62
11	1st–11th	97%	50%	53%	83%	0.71
12	1st–12th	92%	40%	37%	76%	0.6
13	1st–13th	92%	80%	47%	86%	0.77
14	1st–14th	93%	72%	42%	85%	0.75
15	1st–15th	92%	49%	85%	82%	0.75
16	1st–16th	90%	83%	63%	86%	0.77
17	1st–17th	89%	88%	0%	82%	0.55
18	1st–18th	92%	78%	65%	86%	0.80
19	1st–19th	96%	61%	0%	81%	0.51
20	1st–20th	91%	90%	45%	87%	0.79
Average		94%	52%	29%	81%	0.59
